# Prognosis of Patients With Colorectal Cancer and Apical Lymph Node Metastasis at the Inferior Mesenteric Artery: A Systematic Review and Meta-Analysis

**DOI:** 10.3389/fmed.2021.800717

**Published:** 2022-01-17

**Authors:** Senjun Zhou, Yi Shen, Chen Huang, Gang Li

**Affiliations:** Department of Colorectal and Anal Surgery, Shaoxing People's Hospital, Shaoxing, China

**Keywords:** apical lymph node, meta-analysis, metastasis, overall survival, apical lymph node metastasis

## Abstract

**Background:**

This review was designed to compile the evidence on the prognosis of patients with colorectal cancer and apical lymph node (APN) metastasis and the long-term benefit of inferior mesenteric artery lymph node (IMA-LN) resection.

**Methods:**

We searched the PubMed Central, Cochrane library, EMBASE, and MEDLINE databases from inception until May 2021 for relevant publications. We assess the quality of the studies using the Newcastle Ottawa scale. We conducted a random-effects model meta-analysis and report pooled hazard ratios (HRs) with 95% confidence intervals (CIs).

**Results:**

We analyzed data from 13 studies conducted in Japan, China, and Korea with 6,193 participants. Most studies were retrospective in nature and of low quality. We found that patients with APN metastasis had shorter OSs (pooled HR, 2.41; 95% CI, 1.92–3.02) and PFSs (pooled HR, 2.42; 95% CI, 1.90–3.09) than the patients without the metastasis. We identified significant heterogeneity without publication bias for both outcomes. Moreover, our sensitivity analysis revealed robust estimates were robust for the individual effects.

**Conclusion:**

Our findings suggest that patients with colorectal cancer and APN metastases have significantly worse OS and DFS than those without the metastasis. However, inclusion of low-quality retrospective studies with high heterogeneity limits the generalizability of study findings.

## Introduction

Inferior mesenteric artery (IMA) lymph nodes (LNs), also known as the apical lymph nodes (APN), are situated between the IMA and left colonic artery origins (1, 2). They collect the lymphatic drainage of the left colon and rectum. The APN are classified as third station lymph nodes across the sigmoid colon or rectum (1, 2). APN have been studied as the site of metastases and curative ligations in colorectal cancer (3). Colorectal surgeons are aware of the possible association between the anastomosis leak rate and the prognosis after IMA ligation (high ligation/low ligation). However, the oncological and prognostic value of APN remains unclear, and the usefulness of APN resection is still debated and has been challenged by surgeons worldwide (3).

The Dukes' classification considers the involvement of the APN around the IMA origin in rectal cancer. Moreover, APN may presage systemic metastases and are an indication for D3 radical excision. Few reports have dealt with the prognosis or the recurrence patterns amongst patients with colorectal cancer and APN metastasis (3), probably due to the relatively low incidence of the metastasis. Studies have shown that the frequency of APN resections does not affect the survival and that the procedure may even increase the risk of pelvic plexus injury and hemorrhage (4–8). Despite these debates, the prognosis of patients with colorectal cancer and APN metastasis and the evidence for the potential long-term benefit of APN lymphadenectomy remains unclear. Hence, we systematically searched the literature and conducted a review and meta-analysis to synthesize the relevant evidence.

## Materials and Methods

### Eligibility Criteria

#### Study Design

We included relevant full-text randomized controlled trials (RCTs) and observational studies (retrospective/prospective) and excluded conference abstracts, unpublished data, and gray literature.

#### Participants

We analyzed data from patients with colorectal cancer.

#### Exposure

We included studies reporting relevant outcomes in patients with (positive) and without (negative) APN metastasis.

#### Outcomes

We included studies with overall survival (OS) and disease-free survival (DFS) results.

### Search Strategy

We systematically searched PubMed Central, EMBASE, MEDLINE, and Cochrane library electronic databases for entries in English from database inception to May 2021 after selecting medical subject headings (MeSH) and free-text words during the protocol stage. We searched the engines and databases including appropriate truncations, wildcards, and proximity searching of keywords and their synonyms using combinations of the following terms: “Apical Lymph Node,” “Apical Lymph Node Metastasis,” “Nodal Metastasis,” “Colon Cancer,” “Mortality,” “Overall Survival,” “Disease-Free Survival,” “Rectal Cancer,” “Relapse-Free Survival,” “Colorectal Cancer.” In addition, we looked for key concepts with relevant subject headings. We used “OR” and “AND” Boolean operators to combine individual search results and study type filters to narrow down our final search. We also manually searched the reference lists of the articles retrieved to identify relevant missing publications.

### Study Selection Process

**Figure 1:**
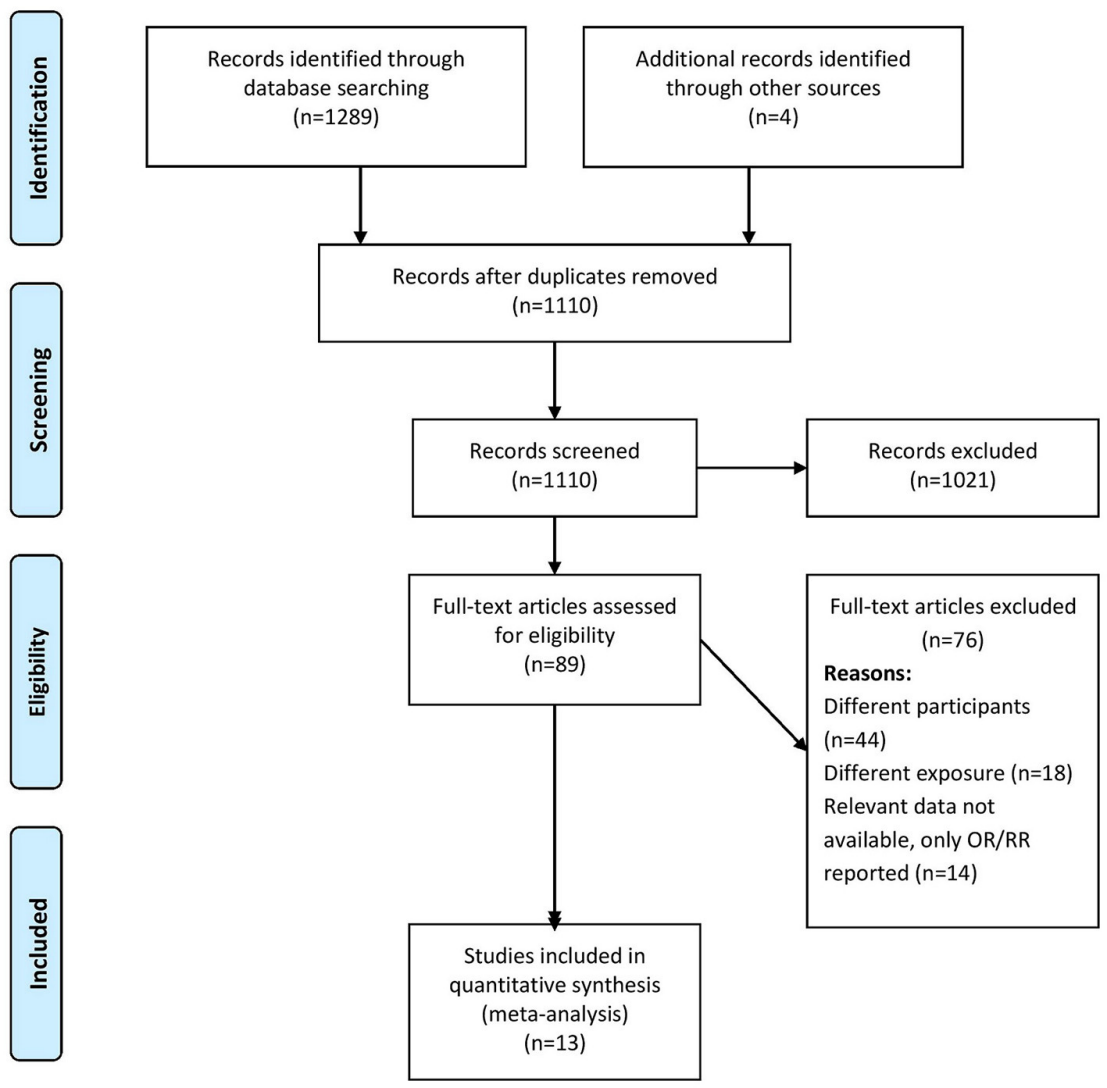
PRISMA flowchart (*n* = 13).

Two independent researchers (SZ and YS) screened the titles, abstracts, and keywords and imported the citations, titles, and abstracts to an Endnote library to prepare a final list for a secondary screen for inclusion after removing duplicates. They obtained full-texts for the resultant studies and screened them against our eligibility criteria. We excluded studies not satisfying the eligibility criteria and noted the relevant reasons. Discussions with a third researcher (CH) served to reach a consensus in cases of disagreements during the screening process. Figure 1 shows the screening and selection process in a PRISMA flow chart.

### Data Collection Process and Management

We designed a data extraction form to classify general study information including its publication year, title, study design, sample size, participants, inclusion and exclusion criteria, outcome assessment methods, mean age, cancer staging, quality-related information, and outcome-related information (OS and DFS). The primary researcher (SZ) collected and entered the data into the form, and secondary researchers (CH and GL) double-checked the correctness of entries.

### Risk of Bias Assessment

Two independent researchers (CH and GL) used the Newcastle Ottawa (NO) Quality Assessment Form for observational studies to assess bias under three domains: selection, comparability, and outcome (9). We graded the risk for each domain as low (adequate information provided), high (inadequate information or “not performed”), or unclear (missing information), and we considered studies with a score of five or higher in the NO scale as being of high quality.

### Statistical Analysis

We used STATA version 14.2 (StataCorp, CollegeStation, TX, USA) to perform the meta-analysis. We calculated pooled estimates of OS and DFS using the natural hazard ratios logarithm [ln(HR)] and its standard error. We initially extracted the HRs with 95% confidence intervals (CIs) from the studies included and then calculated their natural logarithms and the corresponding standard errors (10). We used lower and upper confidence HR limits and a cumulative distribution function of the normal distribution to obtain their logarithmic variance:


$$\begin{array}{r} Variance\;[ln(HR)]\;=\;[ln(upper\;CI\;of\;HR)-ln(lower\;CI\;of\;HR)\\ \;/2\times \;\mathrm{1.96}] \end{array}$$

We calculated the square root of the logarithmic HR variance to obtain the corresponding standard error (SE):


SE[ln(HR)]=√Variance [ln(HR)]

We estimated pooled effects in STATA after entering the logarithmic HR and SE values obtained. We anticipated a large study heterogeneity and chose a random effects model accordingly. The final data are expressed as pooled HRs with 95% CIs for OS and DFS. We generated a forest plot for a visual representation of the pooled estimates.

We assessed the heterogeneity by obtaining chi square *I*^2^ statistic values. Chi square test *P* ≤ 0.10 were as indicative of significant heterogeneity, and *I*^2^ values were indicative of mild (<25%), moderate (25–75%), or substantial (>75%) heterogeneity. Subgroup analyses and a meta-regression helped us explore heterogeneity causes. We generated a funnel plot to visualize and assess publication bias evaluating the asymmetry of the plot using Egger's test. We considered publication bias *P* < 0.10 as statistically significant. We identified individual study effects and the robustness of results with a sensitivity analysis.

## Results

### Study Selection

Figure 1 shows PRISMA flowchart representing our study selection process. We retrieved 1,289 records and 85 full-text studies after the initial screening. We conducted a secondary screening including these studies and four more retrieved from their reference lists. Finally, we analyzed data from 13 studies satisfying the inclusion criteria (*n* = 6,193 participants) (3, 11–22).

### Study Characteristics

Most studies (11 out of 13) were retrospective in nature, and most were conducted in Japan (*n* = 4) and China (*n* = 4). The mean ages of participants ranged from 60 to 71.2 years. The sample sizes amongst the studies varied from 97 to 1,205 participants. The median follow-up lengths ranged from 22 months to 6.1 years. Most studies (*n* = 8) were conducted among patients with stage III colorectal cancer. We found 11 studies reporting DFSs and 10 reporting OS (Table 1). We found nine poor quality studies; the rest were of high quality (Table 2).

**Table 1:** Characteristics of the included studies (*N* = 13).

**Study No**	**References**	**Country**	**Study design**	**Sample size**	**Stage**	**Follow-up**	**Outcomes**	**Type of**	**Mean age**	**Quality of**
						**duration**	**reported**	**analysis**	**(in years)**	**evidence**
1.	Ang et al. (11)	UK	Retrospective	242	Duke Stage C	NR	Overall survival	MVA	70	Low
2.	Chen et al. (15)	China	Retrospective	578	Non-metastatic CRC	Median = 37.4 months	Disease free survival	MVA	60	Low
3.	Huh et al. (13)	Korea	Prospective	1,205	Stage I–IV	Median = 54.2 months	Overall survival and disease-free survival	MVA	62	High
4.	Kang et al. (18)	Korea	Prospective	625	Stage III	Median = 52 months	Disease-free survival	MVA	NR	High
5.	Kim et al. (16)	Korea	Retrospective	187	Stage III	Median = 42.2 months	Overall survival and disease-free survival	MVA	71.2	Low
6.	Kobayashi et al. (14)	Japan	Retrospective	485	Stage III	Mean = 6.1 years	Overall survival	MVA	62	Low
7.	Nagasaki et al. (17)	Japan	Retrospective	446	Stage III	Median = 60.2 months	Disease-free survival	MVA	66	High
8.	Peng et al. (19)	China	Retrospective	510	Stage III	Median = 47 months	Overall survival and disease-free survival	MVA	NR	Low
9.	Rao et al. (21)	China	Retrospective	801	Stage I–IV	Median = 34 months	Overall survival and disease-free survival	MVA	64	Low
10.	Tsai et al. (11)	Taiwan	Retrospective	97	Stage III	Median = 22 months	Overall survival and disease-free survival	MVA	64	High
11.	Wang et al. (20)	Japan	Retrospective	498	Stage III	NR	Overall survival and disease-free survival	MVA	64	Low
12.	Wang et al. (22)	Japan	Retrospective	254	Stage III	NR	Overall survival and disease-free survival	MVA	70	Low
13.	Zhao et al. (3)	China	Retrospective	265	Stage I–IV	Median = 27 months	Overall survival and disease-free survival	MVA	62	Low

*NR, Not reported; UK, United Kingdom; CRC, Colorectal cancer; MVA, Multivariable Analysis*.

**Table 2:** Quality assessment of the included studies (*N* = 13).

**S.N.**	**References**	**Representativeness**	**Sample size**	**Non-**	**Ascertainment**	**Control for**	**Assessment of**	**Statistical**	**Overall**
			**justification**	**response**	**of exposure**	**confounding**	**outcome**	**tests**	**Quality**
1	Ang et al. (11)	0 star	0 star	0 star	0 star	^**^	0 star	^*^	Low
2	Chen et al. (15)	0 star	0 star	0 star	0 star	^**^	0 star	^*^	Low
3	Huh et al. (13)	^*^	0 star	^*^	^*^	^**^	^*^	^*^	High
4	Kang et al. (18)	^*^	^*^	0 star	^*^	^**^	^*^	^*^	High
5	Kim et al. (16)	0 star	0 star	0 star	0 star	^**^	^*^	0 star	Low
6	Kobayashi et al. (14)	0 star	0 star	0 star	0 star	^**^	^*^	^*^	Low
7	Nagasaki et al. (17)	^*^	^*^	^*^	0 star	^**^	^*^	^*^	High
8	Peng et al. (19)	^*^	0 star	0 star	0 star	^**^	0 star	^*^	Low
9	Rao et al. (21)	0 star	0 star	^*^	0 star	^**^	^*^	0 star	Low
10	Tsai et al. (11)	^*^	0 star	^*^	^*^	^**^	0 star	^*^	High
11	Wang et al. (20)	0 star	0 star	0 star	0 star	^**^	^*^	^*^	Low
12	Wang et al. (22)	0 star	0 star	^*^	^*^	^*^	0 star	^*^	Low
13	Zhao et al. (3)	0 star	0 star	0 star	^*^	^**^	0 star	^*^	Low

*Represents the number of points given for each domain to do the risk of bias scoring;

* *one point;*

** *two points*.

### Overall Survival

**Figure 2:**
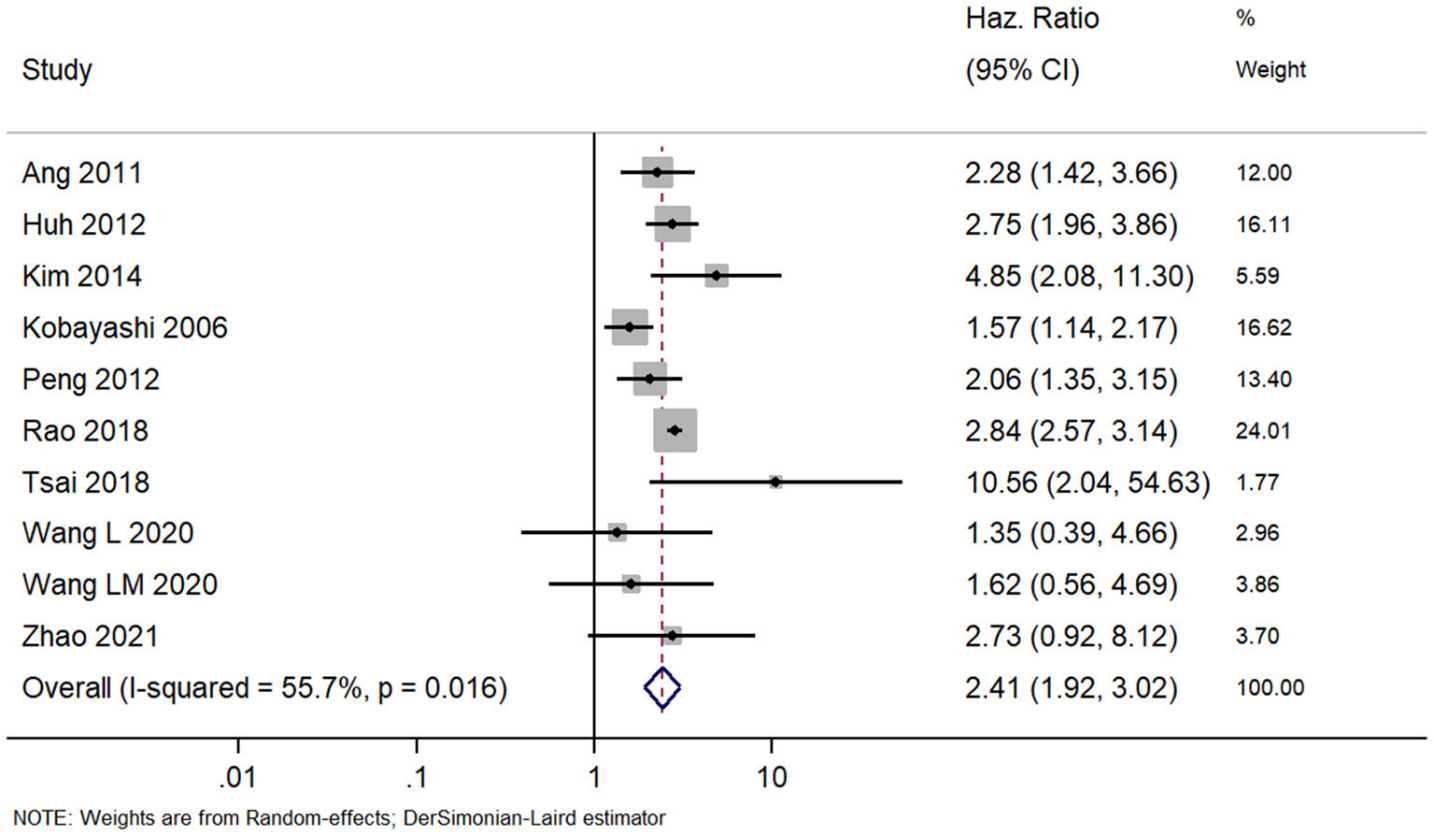
Forest plot showing the difference in overall survival between the APN-positive and -negative patient cohorts (data from 10 studies).

In total, 10 studies reported prognoses of patients with colorectal cancer and APN metastasis. The pooled HR was 2.41 (95% CI, 1.92–3.02) indicating that the patients with positive APN have significantly worse survivals than those with negative APN (Figure 2). However, the high heterogeneity identified among the studies (*I*^2^ = 55.7%; chi square test for heterogeneity, *p* = 0.02) prompted us to explore its sources. We used meta-regression with potential covariates (study design, country, sample size, mean age, follow-up duration, stage of disease, site of cancer, and quality of studies) and found that only the study country had a statistically significant value that explained ~81.5% of the total heterogeneity (reflected on the between-study variability).

**Figure 3:**
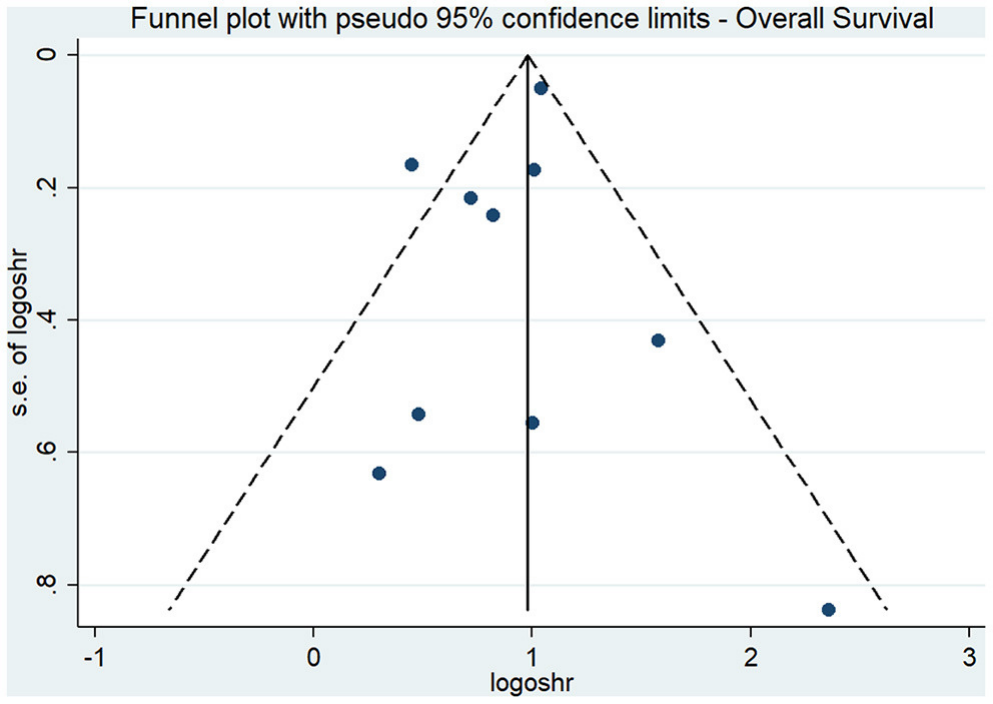
Funnel plot for overall survival.

**Figure 4:**
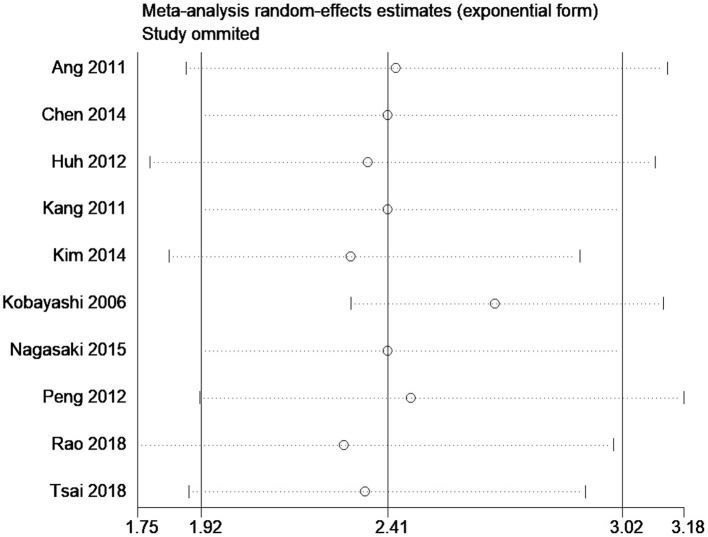
Sensitivity analysis for overall survival.

The results of our subgroup analyses for the study designs (prospective/retrospective), sample sizes (< or >300 patients), quality of studies (low or high), and country (Japan/China/Korea/United Kingdom/Taiwan) showed similar effect estimates in terms of study design (prospective HR, 2.75; 95% CI, 1.96–3.86; *n* = 1; and retrospective HR, 2.36; 95% CI, 1.79–3.11; *n* = 9), and sample sizes (<300 sample size pooled HR, 2.94; 95% CI, 1.81–4.78; *n* = 5; and >300 sample size pooled HR, 2.24; 95% CI, 1.69–2.97; *n* = 5). However, the study quality (low quality pooled HR, 2.27; 95% CI, 1.73–2.96; *n* = 8; and high quality pooled HR, 4.20; 95% CI, 1.23–14.27; *n* = 2) and study country (China pooled HR, 2.76; 95% CI, 2.44–3.13; *n* = 3; Japan pooled HR, 1.56; 95% CI, 1.15–2.11; *n* = 3; Korea pooled HR, 3.18; 95% CI, 1.96–5.17; *n* = 2) variables both exhibited high between-study variability. The funnel plot (Figure 3) did not reveal publication bias evidence (Egger's test *p* = 0.49), and the sensitivity analysis (Figure 4) showed similar effect estimates' magnitude and direction due to individual study effects.

### Disease-Free Survival

**Figure 5:**
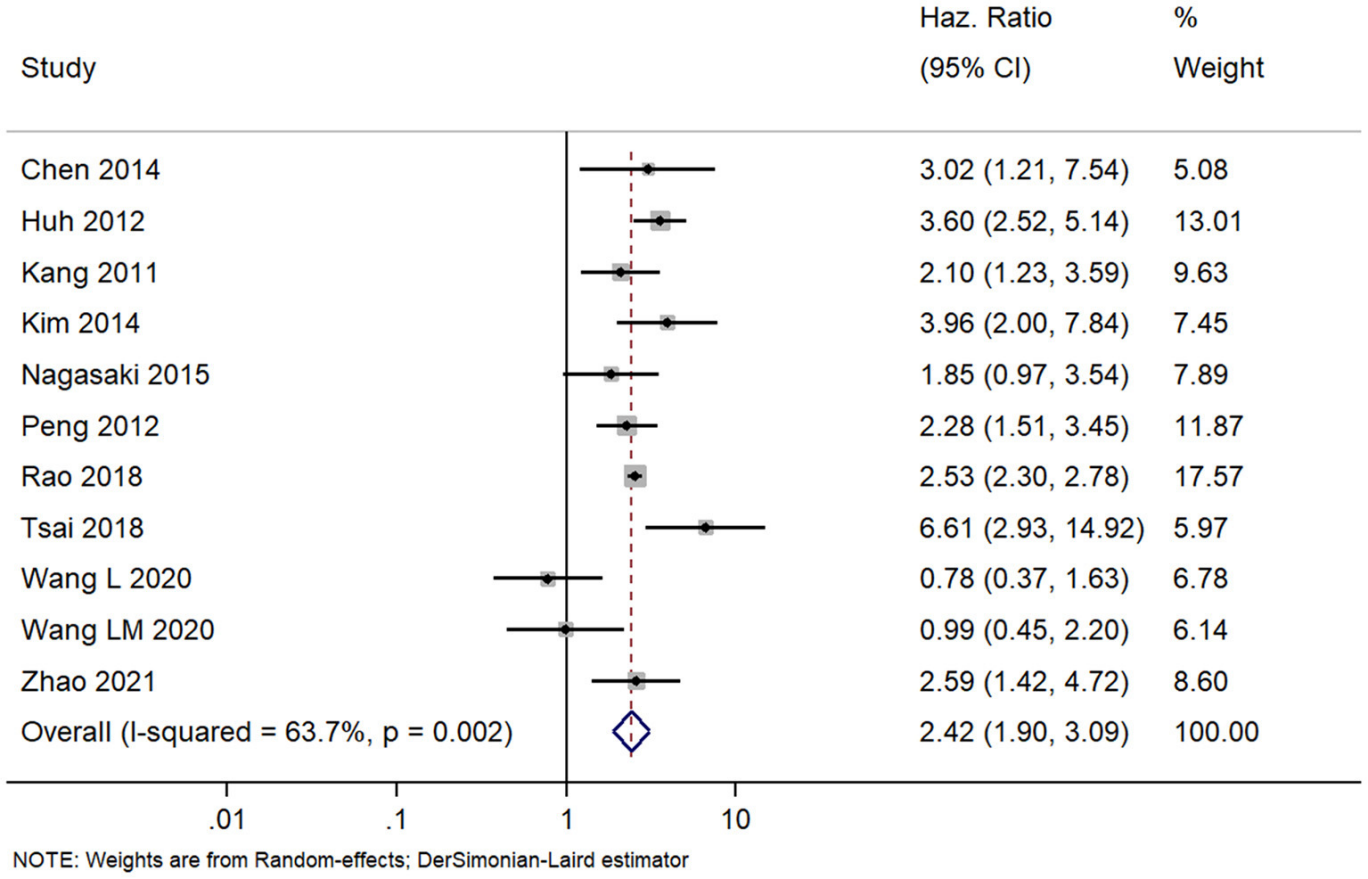
Forest plot showing the difference in disease-free survival between the APN-positive and -negative patient cohorts (data from 11 studies).

We found 11 studies with prognoses of patients with colorectal cancer and APN metastasis. The pooled HR was 2.42 (95% CI, 1.90–3.09) indicating that the patients with positive APN have significantly worse DFSs than those with negative APN (Figure 5). However, we found high heterogeneity among the studies (*I*^2^ = 63.7%; chi square test for heterogeneity, *p* = 0.002). We explored the heterogeneity sources based on meta-regression with the same set of covariates we used for the analysis of OS. Here also, only the country and stage of disease had a statistically significant value and was able to explain the total heterogeneity.

**Figure 6:**
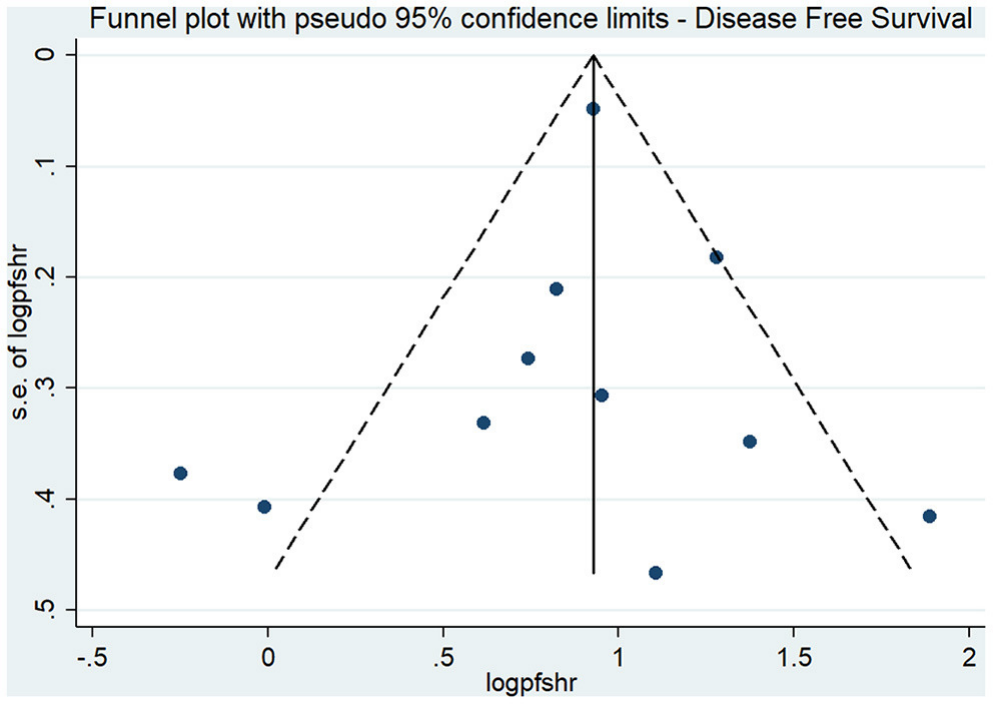
Funnel plot for disease-free survival.

**Figure 7:**
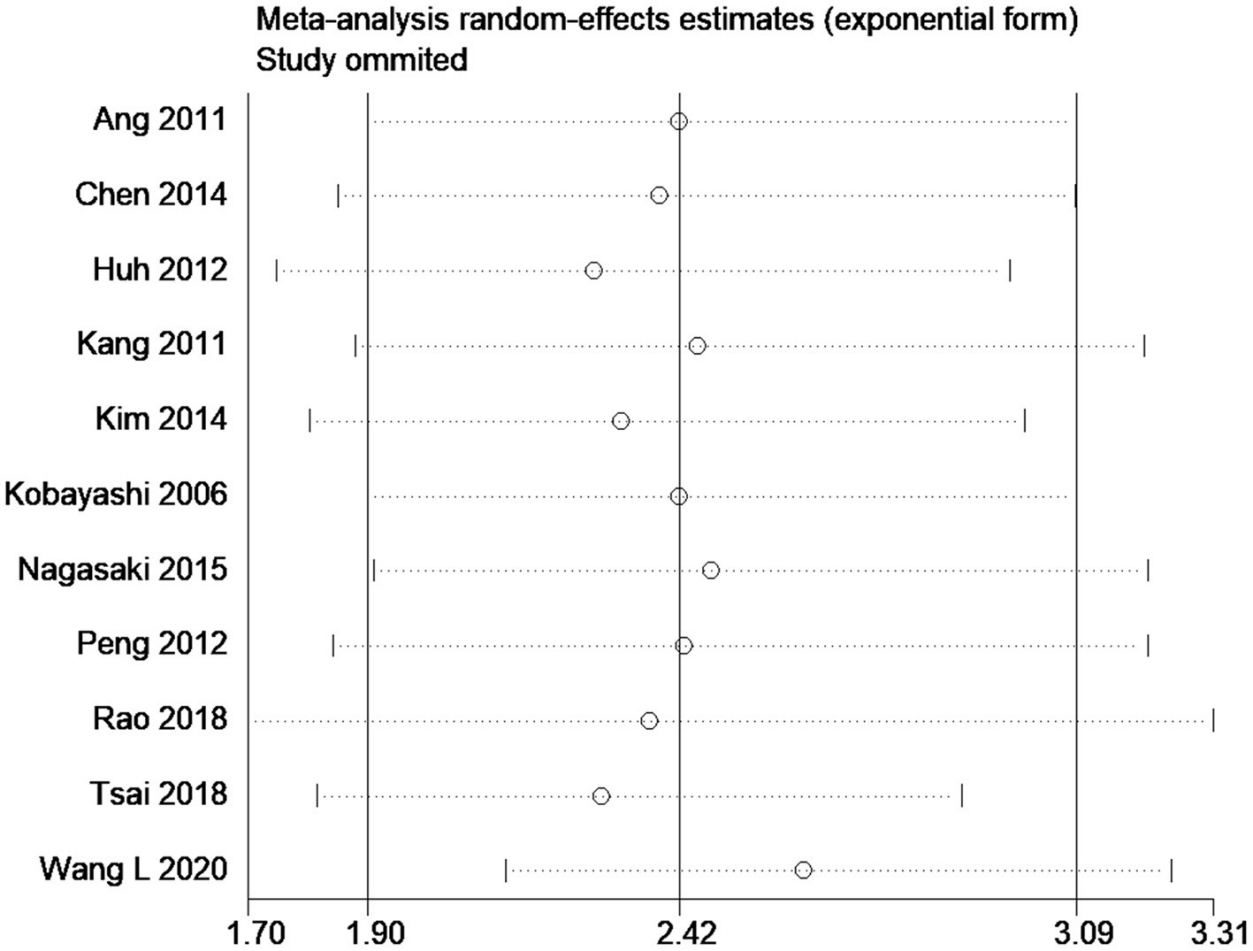
Sensitivity analysis for disease-free survival.

The effect estimates after our subgroup analysis for study design (prospective HR, 2.85; 95% CI, 1.69–4.82; *n* = 2; and retrospective HR, 2.30; 95% CI, 1.70–3.10; *n* = 9), sample sizes (<300 sample size pooled HR, 2.86; 95% CI, 1.40–5.84; *n* = 4; and >300 sample size pooled HR, 2.30; 95% CI, 1.78–2.97; *n* = 7), and study quality (high quality pooled HR, 3.00; 95% CI, 1.87–4.80; *n* = 4; and low-quality pooled HR, 2.13; 95% CI, 1.55–2.93; *n* = 7) were all similar. However, we found significant country variations (China pooled HR, 2.52; 95% CI, 2.30–2.76; *n* = 4; Japan pooled HR, 1.16; 95% CI, 0.68–1.98; *n* = 3; Korea pooled HR, 3.12; 95% CI, 2.16–4.49; *n* = 3). Figure 6 shows a funnel plot without evidence of publication bias (Egger's test *p* = 0.72). The magnitude and direction of the effect estimates (due to individual study effects) were similar in the sensitivity analysis (Figure 7).

## Discussion

The location of metastatic lymph nodes has been studied widely across Asian countries as per Japanese general rules for the clinical and pathological study of colon, rectum, and anal region cancers (23). The prognostic importance of the presence or absence of APN metastasis around the IMA remains unclear. A positive APN has been proposed to result in poor survival outcomes (17–20). Therefore, reviewing the available literature and calculating pooled effects on patient outcomes is important. We conducted this review to assess the prognosis of patients with colorectal cancer and APN metastasis on their OS and PFS.

We used data from 13 studies (*n* = 6,193 participants) for our meta-analysis. Most studies were retrospective in nature and most were conducted in Asia (Japan, China, and Korea). Most studies were of low quality. Both OS (pooled HR, 2.41; 95% CI, 1.92–3.02) and PFS (pooled HR, 2.42; 95% CI, 1.90–3.09) were worse for patients with colorectal cancer and positive APN than for those with negative APN (statistically significant association with confidence limit below the null value of 1). These findings indicate that the APN status may provide an accurate nodal staging that can be used for survival prognosis in patients with colorectal cancer. Unfortunately, these findings are not reflected in the recent “8th American Joint Committee on Cancer (AJCC)” guidelines (24). Hence, we stress the importance of considering APN metastases, or lack thereof, as a prognostic indicator for patients with colorectal cancer. However, our review included only observational studies with a significant interstudy heterogeneity. Furthermore, this review findings are in contrast with a recent trial sequential analysis of RCTs that concluded that there were no significant differences in the surgically treated patients who have underwent high vs. low ligation (25).

We found significant chi-square test heterogeneity and *I*^2^ statistic results for the between-study variability of each outcome. This high heterogeneity can be attributed to methodological differences between the studies included (study design, sample size, mean age, stage, and site of disease etc.).

Hence, we performed meta-regression based on the study design, setting, sample size, follow-up length, and study quality; and, the results indicated that the study country affected the estimates significantly. Subgroup analyses based on the country also revealed a significant variation with respect to both outcomes: the patients with colorectal cancer and APN metastases had the poorest survival in the studies conducted in Korea, but their survival was relatively better in the studies conducted across Japan. We found robust results for the quality of the studies, and our sensitivity analysis ruled out any inappropriate individual study's influence on the overall pooled estimates.

However, our results need to be interpreted and inferred with caution, given that the colorectal cancer might develop in the patients with distinct intestinal diseases such as Inflammatory Bowel Diseases (IBD), Microscopic Colitis (MC), and Irritable Bowel Syndrome (IBS) (26). Different microenvironment poses differential risk of colorectal cancer and metastasis in patients with intestinal disorders. However, all these conditions have almost similar signs and/or symptoms. Such diagnostic overlap between IBD and IBS on one side and MC and IBS.

On the other side should be essentially avoided as each condition poses differential metastasis risk and therapeutic management of colorectal cancer patients (26). It is also worth noting the growing role of microRNA (miRNA) in the development of colorectal cancer and its prognosis (27). The aberrant expression of the miRNA-383 has been shown to contribute to the colorectal cancer tumorigenesis. The ability of miRNA-383 to function as a tumor marker was also evaluated and it showed that the miRNA-383 expression level was significantly down regulated in the patients with colorectal cancer (27). Future studies can also explore the role of such potential tumor markers to understand the prognostic significance of colorectal cancer patients.

The causes for the high mortality in patients with nodal metastases approaching the root of the IMA have to be explored to understand and develop appropriate surgical interventions. A recommendation for surgeons to perform D3 dissections along with APN removal requires further review. The prevalence of APN metastasis is relatively low, but benefits of removing the APN have been found especially in patients with micro-metastasis (28). Non-removal of APN might result in recurrence of lymph nodes metastases across the para-aortic region. Longitudinal studies need to assess the factors responsible for the poor survival of patients with APN metastasis, and they should pinpoint the causes for mortality so that corrective measures and specific interventions can be recommended.

Our literature search was comprehensive and extensive to ensure we had identified all relevant publications to date. To the best of our knowledge, this is the first review assessing the prognosis of patients with colorectal cancer and APN metastasis. However, some limitations in our review are undeniable. As most of the included studies had participants with multiple histologic subtypes of cancer like adenocarcinoma, mucinous, and neuroendocrine tumors, it would be difficult to make any conclusion regarding prognosis. The reason for this is that prognosis of patients with colorectal adenocarcinoma will be vastly different compared with patients with colorectal neuroendocrine tumors. Our results cannot be used to infer causal associations between APN metastases and mortality because we included both prospective and retrospective studies. Thus, prospective studies with large sample sizes are needed to be able to issue recommendations. We could identify only the studies conducted in Eastern/Asian countries, which might limit the generalizability of study findings globally and has higher risk of selection bias. Also, many studies in our analysis were of low quality and had high bias risks. However, our subgroup analyses and meta-regression allowed us to identify the study's country as the source of the high heterogeneity we found.

Regardless of the unavoidable limitations, our results are important because they suggest that APN metastases affect the survival of patients with colorectal cancer, and clinicians, and oncologists should consider our preliminary results (based on reliable pooled estimates) when planning patient management strategies. This study also shows the importance and clinically relevant impact of colorectal screening as it has been reported by previous study that a targeted policy of screening and surveillance by colonoscopy will curb the rising incidence of colorectal cancer (29). Finally, we would like to stress upon certain management practices like the impact of potential dietary strategy for the colorectal cancer prevention and improvement in prognosis potentially impacting in a microbiota remodulation. It has been recently reported that Allium constituents were shown to modify the risk of colon cancer and reduce the mortality rates associated with this malignancy (30). Supplementation of garlic or its extracts reduces the number of aberrant crypt foci, which are one of the earliest pre-neoplastic lesions of colon cancer, and risk of colorectal adenomatous polyps. The prevention of precursor lesions' (adenomatous polyps, crypt foci) formation seems to be an effective strategy to provide an early prevention of colon carcinogenesis, as recently reported (30). However, future robust prospective studies with large sample sizes need to confirm our findings and identify treatment targets to improve the outcomes of patients with colorectal cancer.

## Data Availability Statement

Publicly available datasets were analyzed in this study. The names and locations of the data can be found in the article/supplementary material.

## Author Contributions

SZ and YS conceived and designed the study. CH and GL were involved in literature search, data collection, and analyzed the data. SZ wrote the paper. YS reviewed and edited the manuscript. All authors have read and approved the final manuscript.

## Funding

This work was supported by Shaoxing Science and Technology Plan Project (2018C30073) and Youth Fund Project of Shaoxing People's Hospital (2018YB17).

## Conflict of Interest

The authors declare that the research was conducted in the absence of any commercial or financial relationships that could be construed as a potential conflict of interest.

## Publisher's Note

All claims expressed in this article are solely those of the authors and do not necessarily represent those of their affiliated organizations, or those of the publisher, the editors and the reviewers. Any product that may be evaluated in this article, or claim that may be made by its manufacturer, is not guaranteed or endorsed by the publisher.
